# Estrogen aggravates inflammation in *Pseudomonas aeruginosa *pneumonia in cystic fibrosis mice

**DOI:** 10.1186/1465-9921-11-166

**Published:** 2010-11-30

**Authors:** Yufa Wang, Elvis Cela, Stéphane Gagnon, Neil B Sweezey

**Affiliations:** 1 Physiology and Experimental Medicine, Research Institute, The Hospital for Sick Children, Toronto, Ontario, Canada; 2 Departments of Paediatrics and Physiology, and the Institute of Medical Sciences, University of Toronto, Toronto, Ontario, Canada

## Abstract

**Background:**

Among patients with cystic fibrosis (CF), females have worse pulmonary function and survival than males, primarily due to chronic lung inflammation and infection with *Pseudomonas aeruginosa *(*P. aeruginosa*). A role for gender hormones in the causation of the CF "gender gap" has been proposed. The female gender hormone 17β-estradiol (E2) plays a complex immunomodulatory role in humans and in animal models of disease, suppressing inflammation in some situations while enhancing it in others. Helper T-cells were long thought to belong exclusively to either T helper type 1 (Th1) or type 2 (Th2) lineages. However, a distinct lineage named Th17 is now recognized that is induced by interleukin (IL)-23 to produce IL-17 and other pro-inflammatory Th17 effector molecules. Recent evidence suggests a central role for the IL-23/IL-17 pathway in the pathogenesis of CF lung inflammation. We used a mouse model to test the hypothesis that E2 aggravates the CF lung inflammation that occurs in response to airway infection with *P. aeruginosa *by a Th17-mediated mechanism.

**Results:**

Exogenous E2 caused adult male CF mice with pneumonia due to a mucoid CF clinical isolate, the *P. aeruginosa *strain PA508 (PA508), to develop more severe manifestations of inflammation in both lung tissue and in bronchial alveolar lavage (BAL) fluid, with increased total white blood cell counts and differential and absolute cell counts of polymorphonuclear leukocytes (neutrophils). Inflammatory infiltrates and mucin production were increased on histology. Increased lung tissue mRNA levels for IL-23 and IL-17 were accompanied by elevated protein levels of Th17-associated pro-inflammatory mediators in BAL fluid. The burden of PA508 bacteria was increased in lung tissue homogenate and in BAL fluid, and there was a virtual elimination in lung tissue of mRNA for lactoferrin, an antimicrobial peptide active against *P. aeruginosa *in vitro.

**Conclusions:**

Our data show that E2 increases the severity of PA508 pneumonia in adult CF male mice, and suggest two potential mechanisms: enhancement of Th17-regulated inflammation and suppression of innate antibacterial defences. Although this animal model does not recapitulate all aspects of human CF lung disease, our present findings argue for further investigation of the effects of E2 on inflammation and infection with *P. aeruginosa *in the CF lung.

## Background

Central to the pathogenesis of cystic fibrosis (CF), inflammation and infection (especially by *Pseudomonas aeruginosa *(*P. aeruginosa*)) are mutually reinforcing and eventually lead to respiratory failure, with cor pulmonale as the major cause of death [1,2]. The inflammatory response accounts for the majority of the morbidity and mortality of the disease [3]. Chronic *P. aeruginosa *within the CF airway is a negative determinant of prognosis [4] and the onset of mucoid *P. aeruginosa *colonization is associated with subsequent lung function decline [5,6]. The lungs of CF patients infected with *P. aeruginosa *have increased levels of pro-inflammatory cytokines [7,8] and neutrophils in bronchoalveolar lavage (BAL) fluid [9-11]. Similar findings have been reported in CF mouse models of lung infection with *P. aeruginosa *[12,13].

Helper T-cells (leukocytes that regulate inflammation) were long thought to belong exclusively to either T helper type 1 (Th1) or type 2 (Th2) lineages. However, a distinct lineage named Th17 is now recognized that is induced by interleukin (IL)-23 to produce IL-17 and other pro-inflammatory Th17 effector molecules. Recent evidence suggests a central role for the IL-23/IL-17 pathway in the pathogenesis of CF lung inflammation. Human CF patients with active lung infection with *P. aeruginosa *have elevated sputum levels of IL-23 and of IL-17 [14,15]. Recent studies of adult male mice suggested a role for IL-23, and the Th17 products it induces, in the pathogenesis of murine lung inflammation and neutrophil recruitment in response to airway infection with *P. aeruginosa *[15,16].

Among patients with CF, females have worse survival than males (the so-called "gender gap") [17,18]. Female gender is an important independent risk factor for early detection of mucoid *P. aeruginosa *[19] and for rate of decline in pulmonary function in certain age groups [20]. Females with CF scored worse on a health-related quality of life study [21], and are significantly more likely to have acute pulmonary exacerbations, than their male counterparts [22]. Both wild-type mice and CF transmembrane conductance regulator (CFTR) knockout mice exhibit a female disadvantage in mortality from pneumonia due to a mucoid CF clinical isolate, the *P. aeruginosa *strain PA508 (PA508) [13], and female wild-type mice mount a stronger inflammatory response in their lungs [23].

Although the cause of the CF gender gap has not yet been identified, sex hormones can affect the immune response [24,25]. The female sex hormone 17β-estradiol (E2) has a complex immunomodulatory effect upon inflammation. E2 suppresses acute lung inflammatory responses of mice to lipopolysaccharide-induced injury through an effect on vascular cell adhesion molecules and proinflammatory mediators [26]. However, E2 can also have a proinflammatory role depending on a variety of criteria, as extensively reviewed by Straub [27]. Recent evidence indicates that E2 stimulates T-cell-dependent immune responses [28].

E2 likely contributes to the pathogenesis of the CF gender gap in ways other than direct effects on inflammatory mediators (reviewed by Zeitlin [29]). Coakley et al [30] recently demonstrated that E2 reduces the volume of the airway surface liquid of human CF airway epithelial cells *in vitro*, to a degree that *in vivo *would be expected to interfere with mucociliary clearance, a key component of innate airway defence against infection and inflammation. Chronic infection of CF airway by *P. aeruginosa *is associated with the formation of biofilms [31]. Since neutrophils enhance the formation of *P. aeruginosa *biofilms [32], an activity of E2 to increase neutrophil infiltrates in lung tissue and to increase neutrophils in the BAL fluids would be expected to have a detrimental effect upon CF lungs. E2 may also modulate the formation of *P. aeruginosa *biofilms through an effect upon antimicrobial peptides such as lactoferrin (LTF), a component of innate immunity that interferes with bacterial biofilm development [33].

We tested the hypothesis that E2 aggravates the CF lung inflammation that occurs in response to airway infection with *P. aeruginosa *by a Th17-mediated mechanism. We evaluated the effects of exogenous E2 upon lung infection with PA508 in adult male congenic B6.129P2*^Cftrtm1Unc ^*homozygote mice. We assessed inflammatory cell counts, differential cell counts and bacterial burden in BAL fluid and in lung tissue. Lung tissue inflammation was further assessed using hematoxylin and eosin stained sections and production of mucin was assessed in airways and airway epithelial cells using periodic acid Schiff - stained slides. We also measured BAL fluid levels of inflammatory cytokines and the lung tissue mRNA levels of IL-17A, IL-17F and IL-23, the toll-like receptors 2 and 4 and the antimicrobial peptides LTF (lung tissue) and prolactin-inducible peptide (PIP) in trachea.

## Materials and methods

### Mice

Congenic B6.129P2*^Cftrtm1Unc ^*S489X (null) homozygote male CF knock-out mice [34] were purchased from the Case Western Reserve University's Animal Care Facility, shipped in protective, filtered containers, transported in climate-controlled trucks, and allowed to acclimatize for at least 3 days in the vivarium prior to use. All procedures were approved by the Animal Care Committee of The Hospital for Sick Children, Toronto.

### Injection of Hormone/Vehicle

Mice were injected intraperitoneally (i.p.) with 100 μL of Sesame seed oil (vehicle) with (treatment group) or without (controls) 100 ng of E2 (Sigma, St. Louis, MO) at 10:00 am for six consecutive days, and also at 22:00 pm in the first day.

### PA508 Infection of Mice

On the fifth day of E2 treatment, mice were infected with agar beads impregnated with 1 × 10^6 ^colony forming units (CFUs) of *P. aeruginosa *strain PA 508 in 50 μL directly instilled into the distal trachea using the method of Guilbault *et al. *[13]. Briefly, mice were anesthetized with ketamine and xylazine i.p., placed with the mouth open in a position 30° from vertical on a custom-made restraining board and the tongue pulled aside. The inoculum of infected agar beads was introduced under direct vision into the trachea past the vocal cords using a 24G gavage needle (Harvard Apparatus, Holliston, MA).

PA508, kindly provided by D. Radzioch (McGill University, Montreal, QC), was originally obtained from J Lagacé (University of Montreal, Montreal, QC). This strain has a mucoid appearance when grown on blood agar and was originally isolated from the sputum of a CF patient at Ste-Justine Hospital, Montreal, QC [13,35]. PA 508 was selected to take advantage of its mucoid phenotype and known pathogenicity in human cystic fibrosis airways. Bacteria stocks were stored at -80°C until used.

### Mouse weight

Mice were weighed immediately prior to PA508 infection and just before sacrifice.

### Bronchoalveolar Lavage (BAL)

Immediately post-mortem, lungs were lavaged using 1.0 mL of ice cold 0.9% NaCl. Red blood cells were lysed using ACK lysing buffer [36]. Cells were spun onto glass slides and stained using the Diffquick method following the manufacturer's directions (Protocol^® ^HEMA 3, Fisher Scientific). Differential cell counts were obtained manually under light microscopy. 2-3× 100 cells were counted per slide and means calculated. The supernatant was kept at -80°C until assessed for cytokine content [36].

### Lung Homogenates

Lungs from infected mice were harvested and homogenized with Tenbroeck Tissue Grinders (Wheaton Science Products, Millville, NJ) in 1 mL of sterile PBS per 150 mg of tissue. Lung homogenate was centrifuged at 500 g at 4°C for 10 min and then kept at -80°C until assessed for cytokine content [36].

### Inflammatory Cell counts and Differential Counts

Lungs were excised, minced, and digested for 50 min at 37°C with collagenase D and DNase I (each solution 1 mg/mL, Roche Applied Science). Erythrocytes were lysed using ACK lysing buffer and then the remaining cells were resuspended in staining buffer [37] containing 10% FBS for differential cell counts using flow cytometry. Cells were double stained with PE-anti-CD45 and FITC-anti-Ly6G (Neutrophils), anti-F4/80 (macrophage) (all from BD PharMingen) and fixed with 1.6% paraformaldehyde [37,38]. Labelled samples were analyzed on a FACSCalibur (Becton Dickinson, San Jose, CA). Gating of dead cells was performed using forward light scatter and side light scatter. Analysis of data was performed using FlowJo software (Tree Star, Inc.).

### Lung Histopathology

Lungs were flushed with 0.9% NaCl, slowly inflated with 1 mL of formalin and then completely immersed in formalin. Specimens were embedded in paraffin and 5 μm sections cut. Slides were stained for standard light microscopy using hematoxylin and eosin, periodic acid Schiff (Surgipath, Richmond, IL) and Masson's Trichrome (Sigma, St. Louis, MO) according to the manufacturers' instructions.

### Cytokine Measurements

Lung homogenates and BAL fluid were screened for protein levels of 32 cytokines/chemokines/growth factors (eotaxin, G-CSF, GM-CSF, IFNγ, IL-1α, IL-1β, IL-2, Il-3, IL-4, IL-5, IL-6, IL-7, IL-9, IL-10, IL-12 (p40), IL-12 (p70), IL-13, IL-15, IL-17A, IP-10, KC, LIF, LIX, MCP-1, M-CSF, MIG, MIP-1α, MIP-1β, MIP-2, RANTES, TNFα, VEGF) with the Mouse Cytokine/Chemokine Milliplex™Map kit (Millipore, Billerica, MA) using Luminex™technology according to the manufacturer's instructions, and assayed with the Luminex100IS™system by Linco Research, Inc. The cytokine detection limit for this assay was 3.2 pg/mL.

### Bacterial Burden

Serial dilutions of homogenate and BAL fluid were plated on Petri dishes containing tryptic soy agar. The number of PA508 CFUs was counted after overnight incubation at 37°C.

### RNA Extraction and Real-time RT-PCR

We measured levels of mRNA encoding IL-17A, IL-17F and IL-23, Toll-like Receptor 2 (TLR2), Toll-like Receptor 4 (TLR4) and the antimicrobial peptides LTF in whole lung tissue, and PIP in trachea. Total RNA was extracted from lung or trachea using TRIZOL Reagent (Invitrogen, Carlsbad CA) according to manufacturer's instructions, and reverse transcribed with SuperScript II reverse transcriptase (Invitrogen, Carlsbad CA). Quantitative real-time PCR was performed with the ABI Prism™7900 (Applied Biosystems) using SYBR Green (Eurogentec, San Diego, CA), normalizing all results to the levels of GAPDH mRNA. The following primer sequences were used:

IL-17A, sense 5'-TCCAGAAGGCCCTCAGACTA-3',

anti-sense 5'-AGCATCTTCTCGACCCTGAA-3'.

IL-17F, sense 5'-GTGTTCCCAATGCCTCACTT-3',

anti-sense 5'-GTGCTTCTTCCTTGCCAGTC-3'.

IL-23, sense 5'-GACTCAGCCAACTCCTCCAG-3',

anti-sense 5'-GGCACTAAGGGCTCAGTCAG-3',

LTF, sense 5'-GGAGCCTTGAGGTGTCTGAG-3',

anti-sense 5'-CCAGGTGGCACTCCTTGTAT-3'.

TLR2, sense 5'-TGCTTTCCTGCTGGAGATTT-3',

anti-sense 5'-TGTAACGCAACAGCTTCAGG-3'.

TLR4, sense 5'-GGCAGCAGGTGGAATTGTAT-3',

anti-sense 5'-AGGCCCCAGAGTTTTGTTCT-3'.

PIP, sense 5'-TCCGAAAGCCACTTTTGATT-3'

anti-sense 5'-GTTGAAGGCACCTTCCATTG-3'

GAPDH, sense 5'-GCCATGGACTGTGGTCATGA-3',

anti-sense 5'-TTCACCACCATGGAGAAGGC-3'.

### Statistical Analyses

Data are reported as the mean ± standard error of the mean unless stated otherwise. Using the statistical component of the software package SigmaPlot V11.2 (Jandel Scientific, SPSS Science, Chicago, IL) an unpaired *t*-test was run to compare two different groups unless either of the normality or equal variance tests failed, in which case a Mann Whitney rank sum test was performed. A paired *t*-test was run to compare repeated measures on a single group of individuals at two separate time points. Differences were considered statistically significant when p < 0.05.

## Results

### Mouse weight

Pre-infection weights were not statistically different between control (21.55 ± 1.7 g, n = 6) and E2-treated (23.8 ± 0.9 g, n = 8) mice, p ns. Weights decreased significantly from pre-infection to sacrifice at two days post-infection for both E2-treated (20.96 ± 1.0 g, n = 8, p < 0.05) and control (20.47 ± 1.7 g, n = 6, p = 0.01, paired *t*-test) mice. Weight loss expressed as a percent of pre-infection body weight was significantly greater in E2-treated (11.9 ± 2.2) than in control (5.1 ± 1.3) mice (p < 0.05). None of the infected mice died prior to sacrifice.

### Inflammation

**Figure 1:**
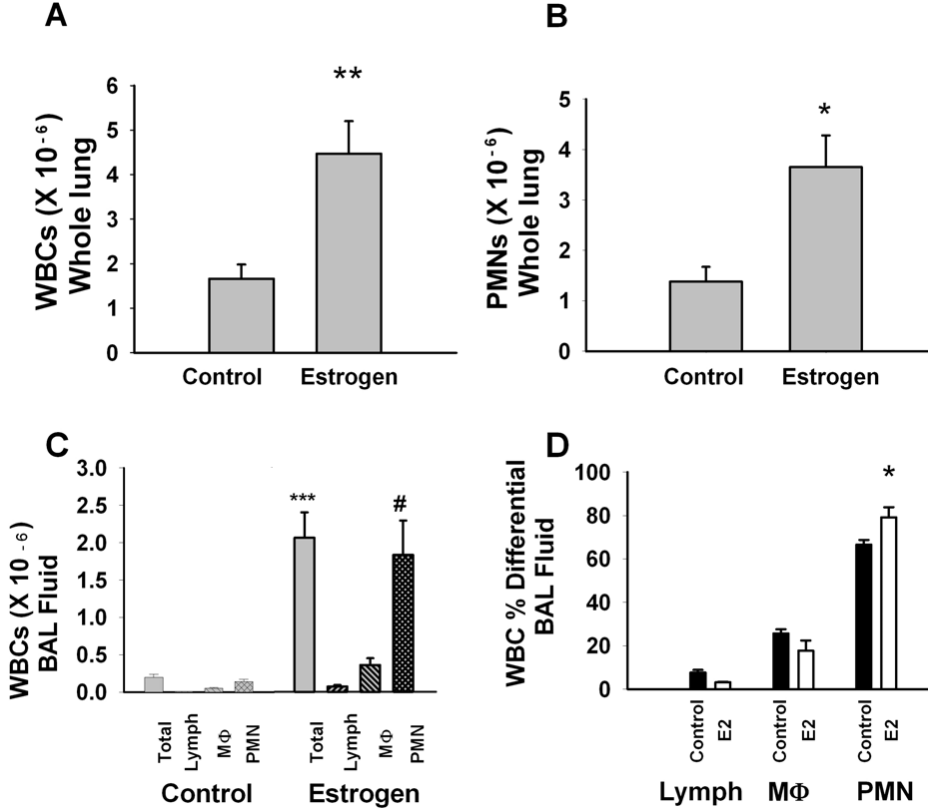
**Estrogen (E2) treatment is correlated with an increase in inflammatory cells**. In whole lung homogenate, (A) Total white blood cells (WBCs), ** p <0.01 vs control, n = 5; and (B) neutrophils (PMNs), * p < 0.05 vs control, n = 5. In bronchoalveolar lavage (BAL) fluid, (C) Absolute cell counts of total WBCs, lymphocytes (lymph), macrophages (M Φ) and PMNs, ** p < 0.0005, # p <0.005, vs control, n = 5; and (D) PMN differential percentage, * p < 0.05 vs control, n = 5.

**Figure 2:**
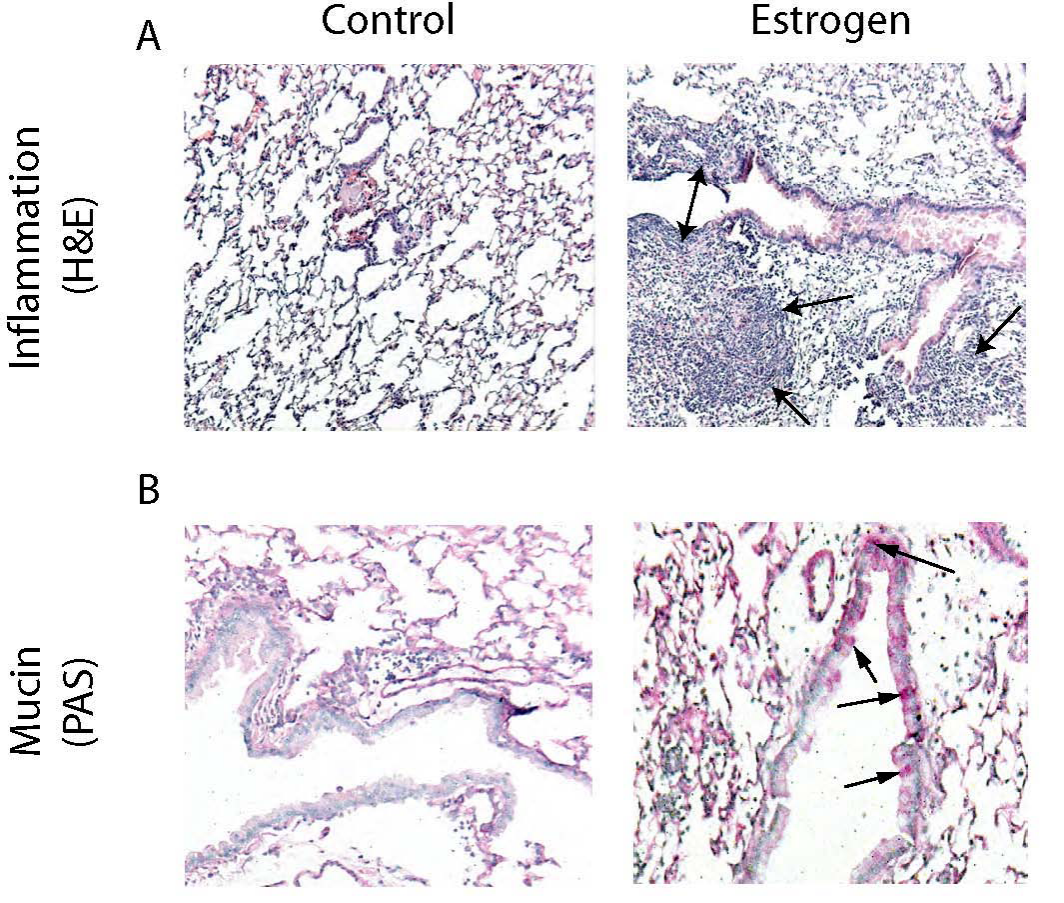
**E2 treatment is correlated with an increase in inflammatory infiltrate and mucin in lung tissue sections**. In lung tissue sections, E2 treated mice had (A) H&E stain: inflammatory infiltrate (arrows) and (B) PAS stain: mucin producing airway lining cells (Pink, arrows). Controls showed much less inflammatory infiltrates or mucin producing cells. Light microscopy, original magnification X 100, n = 4.

Compared to controls, E2-treated mice had significantly higher counts of total white blood cells (WBCs) and of polymorphonuclear cells (PMNs) in both whole lung and BAL fluid (Figure 1). Hematoxylin and eosin (H&E) stained sections of lung tissue revealed a marked inflammatory infiltrate in many areas of E2-treated mice but little or no inflammatory infiltrate was noted in slides from control mice (Figure 2A). Similarly, prominent staining of mucin by Periodic Acid Schiff (PAS) was widely distributed in multiple airway lining cells throughout the large airways of E2-treated mice, staining that was virtually absent in control mice (Figure 2B). However, no difference between groups in collagen deposition was detected upon staining with Masson's Trichrome stain (data not shown).

**Figure 3:**
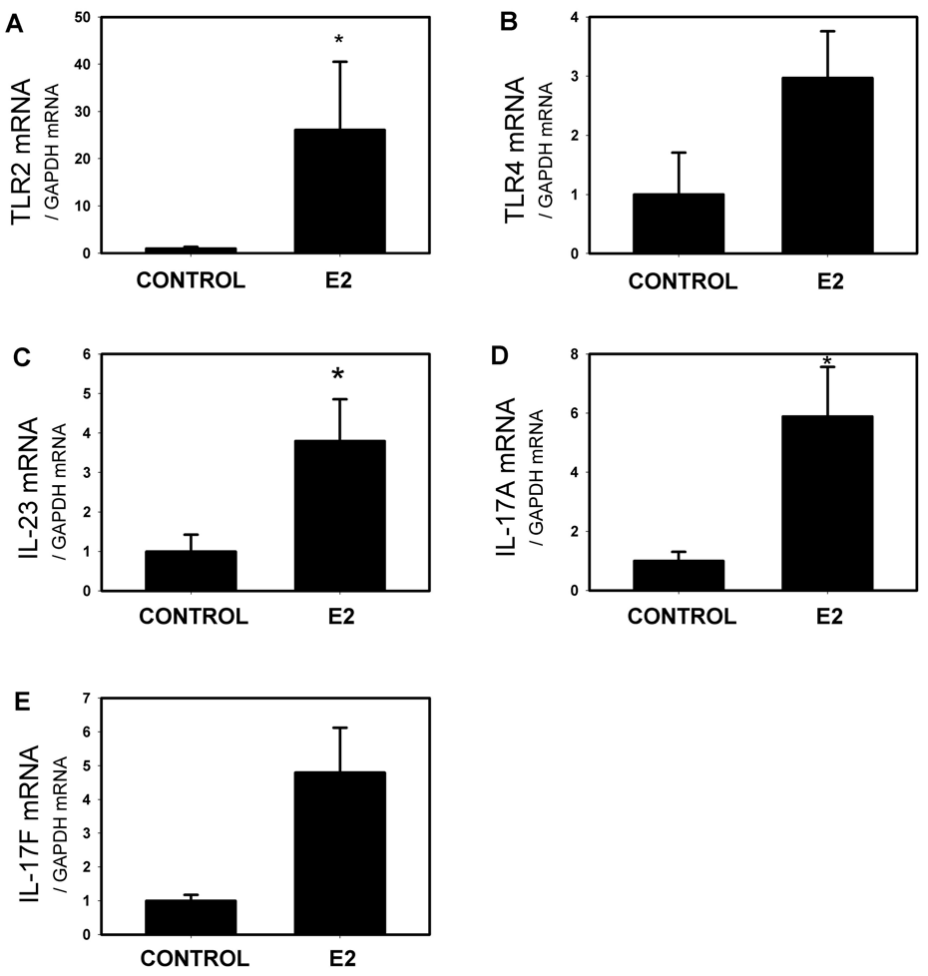
**E2 treatment is correlated with an increase in Toll Like Receptor (TLR) 2 and IL-23/IL17A mRNA levels**. E2 increased (A) TLR2 (* p < 0.05), but not (B) TLR4 mRNA levels (p ns, n = 5). E2 also increased (C) IL-23 and (D) IL-17A (p < 0.05), but not (E) IL-17F (p > 0.05, n = 5)

The toll-like receptors (TLRs) 2 and 4 are important components of the acute, innate inflammatory response of normal and CF airway epithelial cells (reviewed in [39]). In the E2-treated mice, lung tissue mRNA levels for TLR2, but not TLR4, were increased significantly (Figure 3). Levels of mRNA encoding IL-23 and IL-17A, but not IL-17F, were increased (Figure 3).

**Figure 4:**
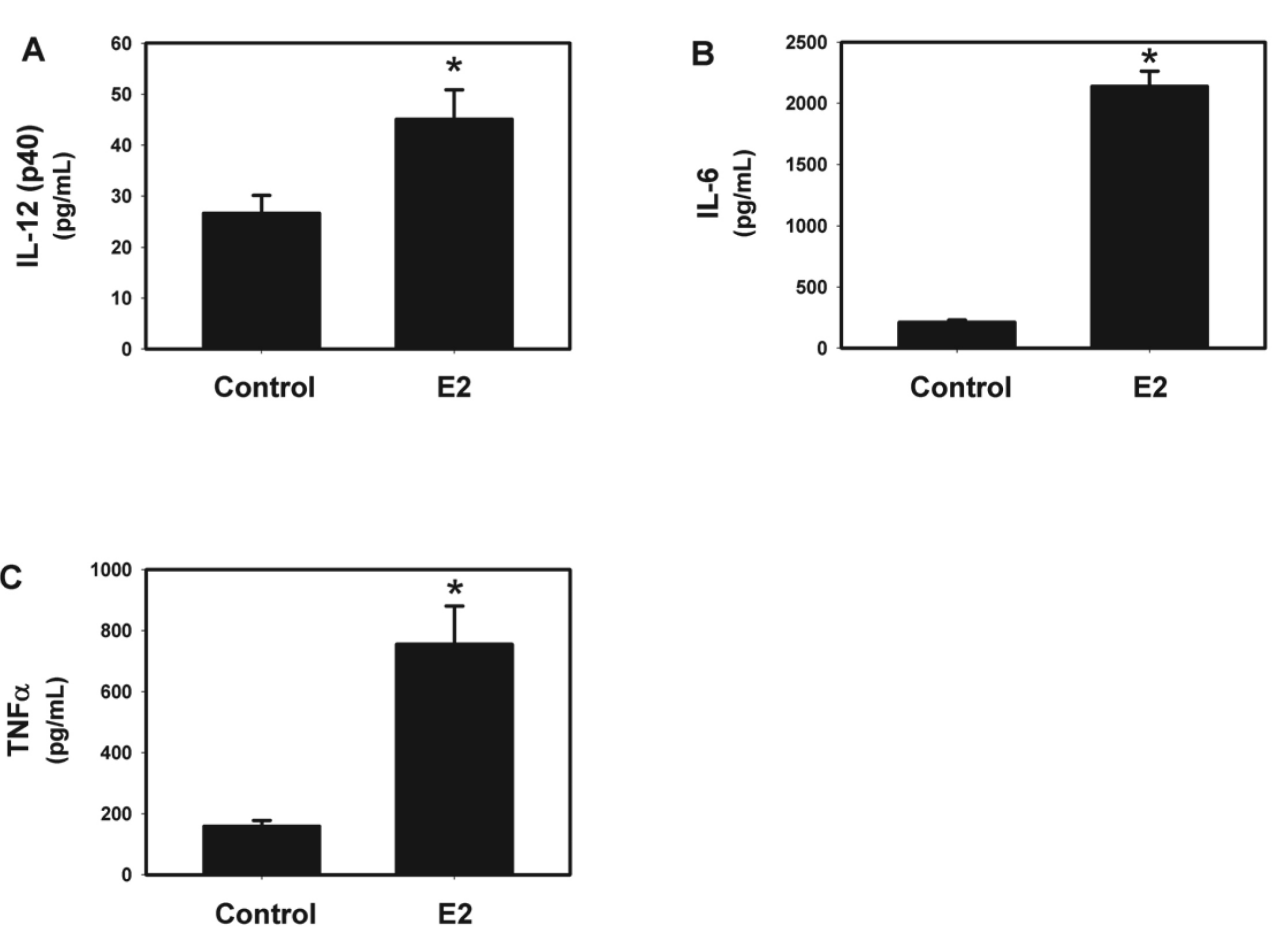
**E2 treatment is correlated with an increase in upstream regulators of Th17 Cells**. E2 increased (A) IL-12(p40), one of two heteromers making up the IL-23 protein, (B) IL-6, and (C) TNFα, important early mediators of acute lung inflammation that induce IL-17. * p < 0.05 vs control, n = 4.

**Figure 5:**
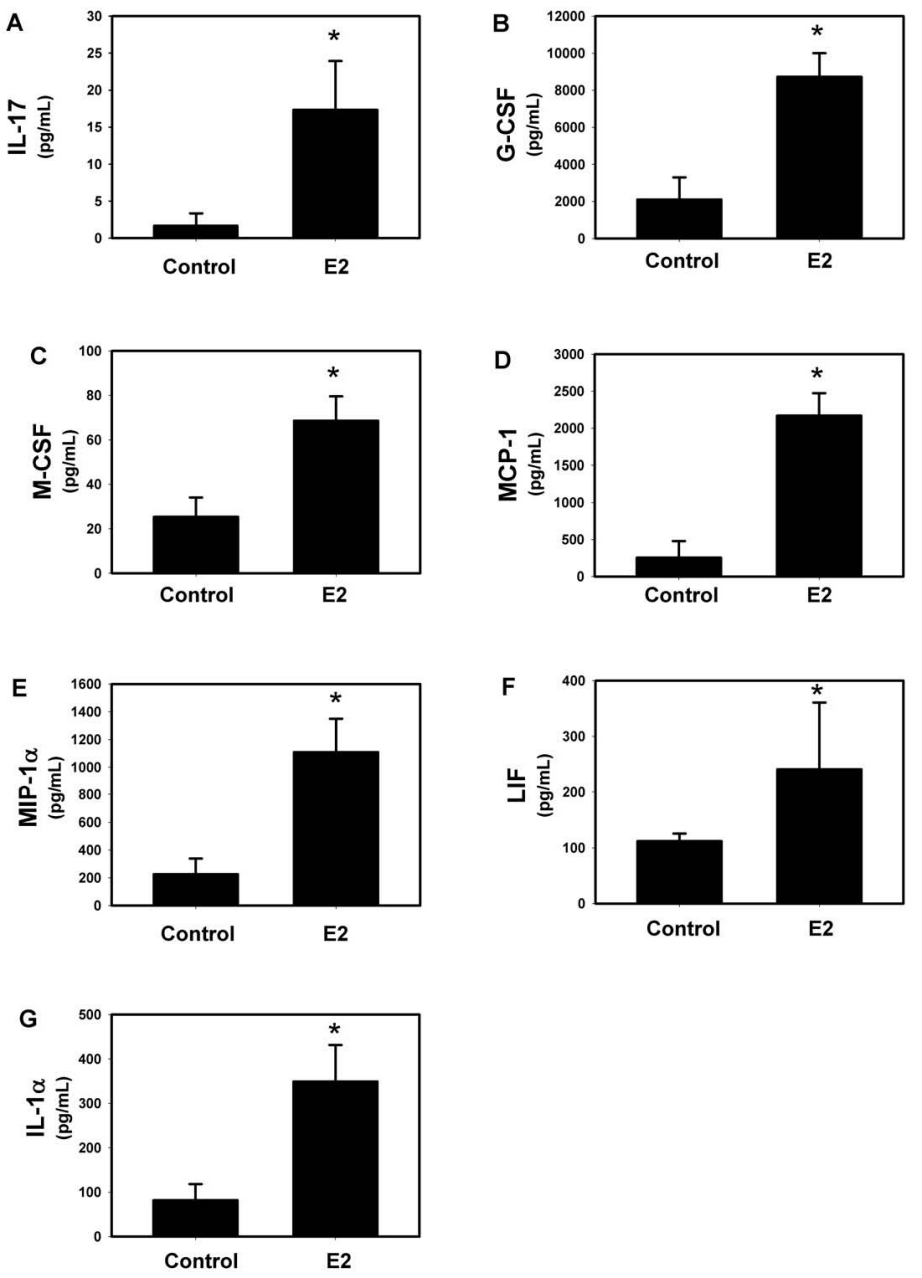
**E2 treatment is correlated with an increase in downstream effectors of Th17 cells**. E2 increased (A) IL-17A, the prototype pro-inflammatory Th17 effector molecule, and a series of downstream effectors known to be modulated by IL-17A, including (B) G-CSF, (C) MCP-1, (D) IL-1α, (E) MIP-1α, (F) LIF, and (G) M-CSF. * p < 0.05 vs control, n = 4.

**Figure 6:**
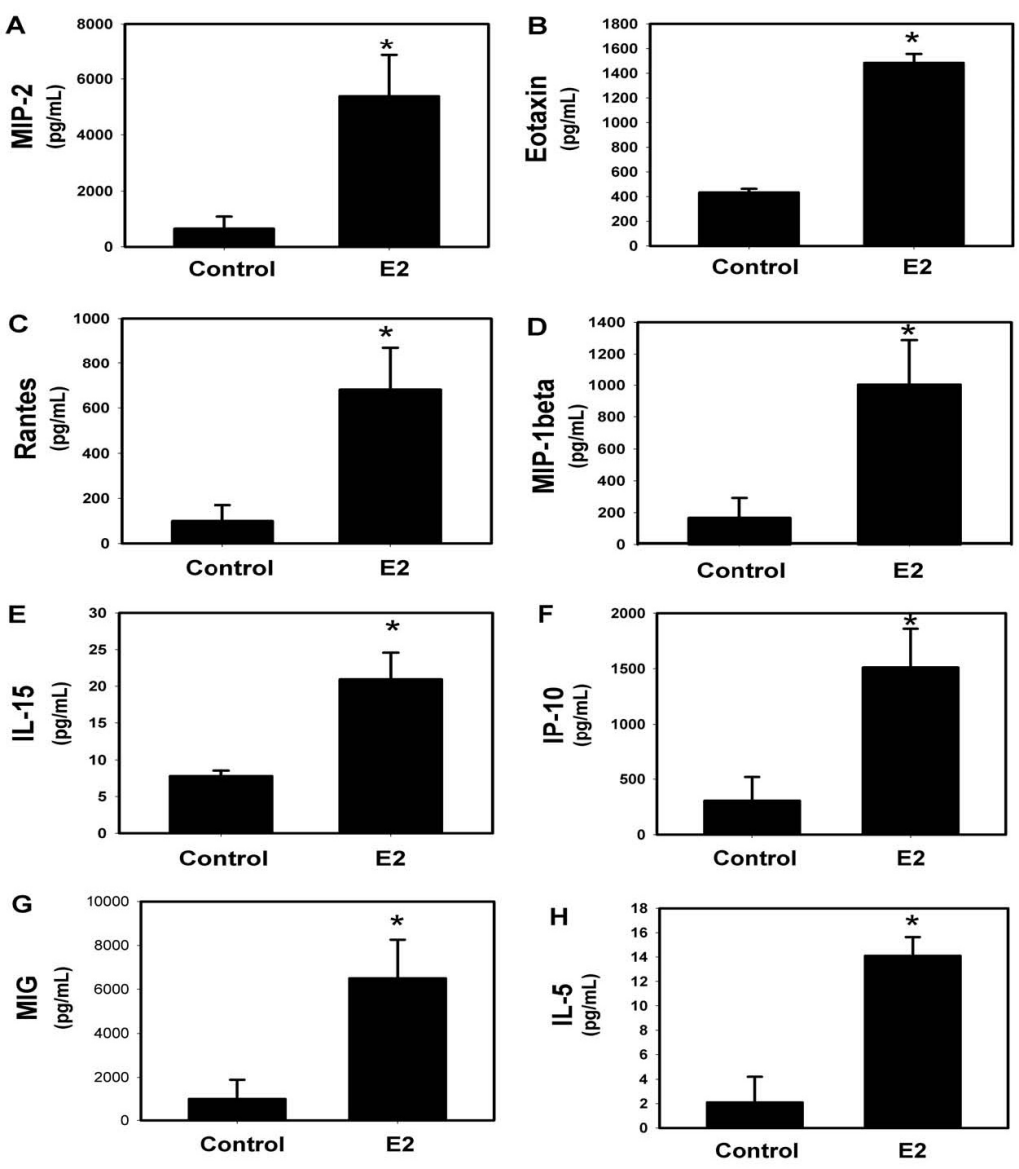
**E2 treatment is correlated with an increase in chemoattractant chemokines**. (A) MIP-2 (recruits neutrophils), (B) eotaxin and (C) RANTES (eosinophils), (D) MIP-1β (macrophages) and (E) IL-15 (mast cells); in CXCR3 chemokines that regulate Th1 cell proliferation, (F) IP-10 and (G) MIG, and the Th2 effector (H) IL-5. * p < 0.05 vs control, n = 4.

**Figure 7:**
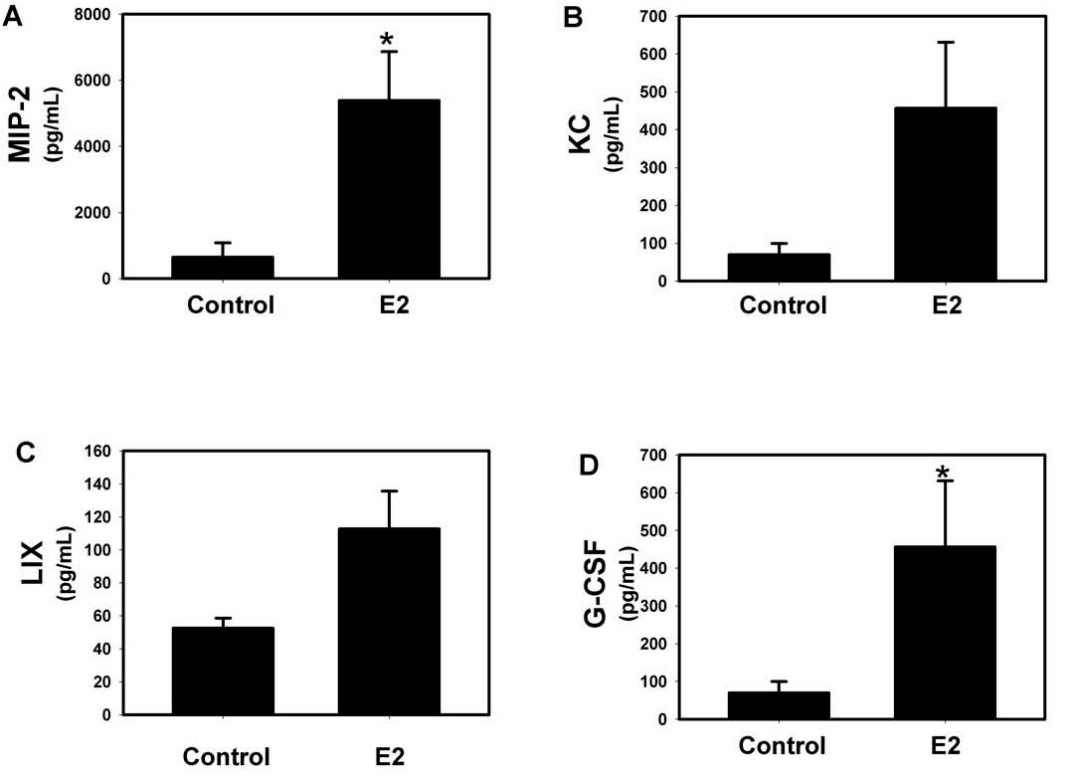
**E2 treatment is correlated with an increase in a murine IL-8 homolog that recruits neutrophils, and in the growth factor G-CSF that stimulates production of neutrophils**. Protein levels of (A) MIP-2, but not (B) KC or (C) LIX, were increased, * p < 0.05, n = 4; (D) G-CSF protein levels were increased, * p < 0.05, n = 4.

IL-23 strongly induces the Th17 pro-inflammatory phenotype in response to mucoid PA508 infection in mice [15]. Therefore, we did a screening measurement of protein levels in BAL fluid of a series of cytokines, chemokines and growth factors, including known upstream modulators of IL-17 and pro-inflammatory Th17 downstream effector mediators. Although measurement of the levels of IL-23 protein is technically inconsistent, levels of IL-12(p40), one of two heteromers making up the IL-23 protein, were increased by E2, as were IL-6 and TNFα, also important early mediators of acute lung inflammation that induce IL-17 (Figure 4). IL-17A itself, the prototype Th17 effector molecule, and a series of downstream effectors that it modulates, were also increased by E2, including G-CSF, MCP-1, IL-1α, MIP-1α, LIF, and M-CSF (Figure 5). A series of chemoattractant chemokines were increased by E2, including MIP-2 (recruits neutrophils), eotaxin and RANTES (eosinophils), MIP-1β (macrophages) and IL-15 (mast cells) (Figure 6). CXCR3 chemokines that regulate Th1 cell proliferation, IP-10 and MIG, and the Th2 effector IL-5 were also increased by E2 (Figure 6). Like MIP-2, KC and LIX are murine IL-8 homologs that recruit neutrophils [40], but after E2 treatment their protein levels did not change to a statistically significant degree (Figure 7). Other protein levels that were assessed but did not change significantly after E2 treatment included IFNγ, IL-1β, IL-3, IL-4, IL-7, IL-9, IL-10, IL-12(p70) (a component of IL-12 that, unlike the p40 component, is not shared with IL-23), and VEGF (data not shown).

### Bacterial Burden

**Figure 8:**
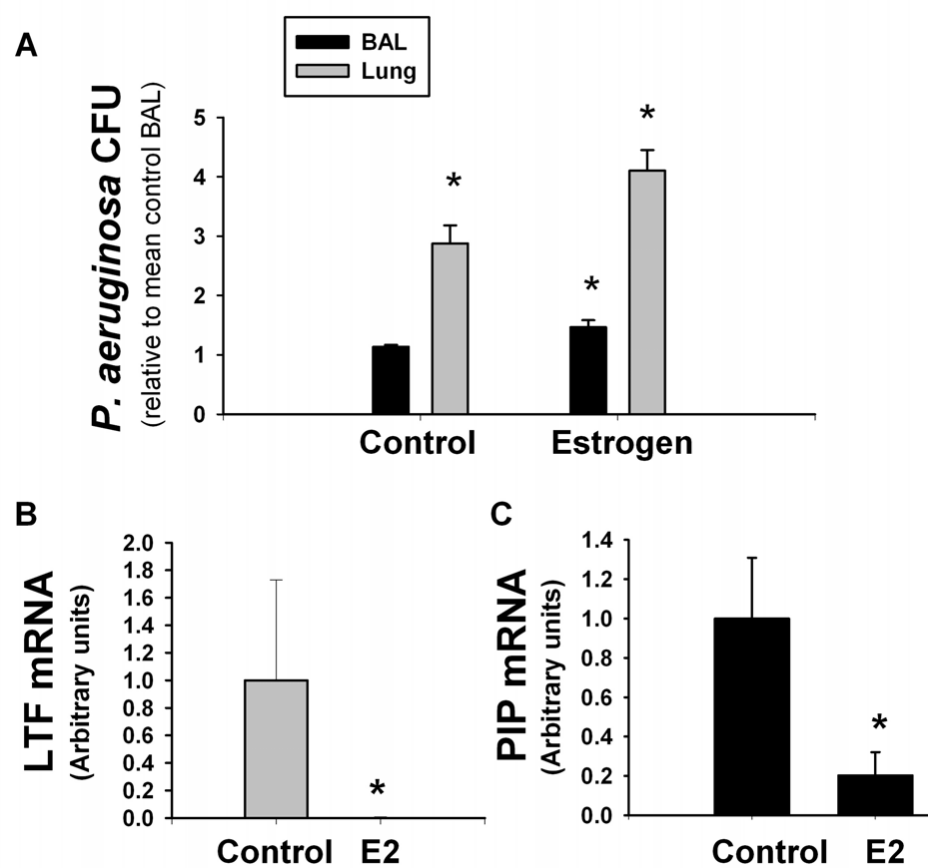
**E2 treatment is correlated with (A) an increase in bacterial burden in whole lung and in BAL fluid, and with a decrease in antimicrobial peptide mRNA levels for (B) LTF and (C) PIP**. * p < 0.05 vs control, n = 5.

E2-treated mice had more PA508 CFUs in whole lung homogenate and in BAL fluid than controls (Figure 8).

### Antimicrobial peptides

Levels of mRNA for the antimicrobial peptides LTF (in lung tissue) and PIP (in trachea), part of the innate immune system, were significantly reduced by E2 treatment (Figure 8). The elimination of LTF mRNA levels upon exposure to E2 was virtually complete, with an average reduction of 200 - fold.

## Discussion

Our present study demonstrates that exogenous E2 exerts a pro-inflammatory effect in a mouse model of CF PA508 pneumonia. This is not surprising, given that both wild-type mice and CFTR knockout mice exhibit a marked female disadvantage in mortality from PA508 pneumonia [13] and female wild-type mice mount a stronger inflammatory response in their lungs [23]. In our adult male CF mice with PA508 pneumonia, E2 increased the numbers of inflammatory cells overall in lung tissue and BAL fluid, with a selective increase in the proportion and total number of neutrophils. Inflammatory infiltrates and mucin production in lung tissue were enhanced and the PA508 bacterial load in both lung tissue and in BAL fluid was increased. These E2-induced changes reproduce the phenotype reported in the female mice in considerable detail, suggesting a central role for E2 in causing the "gender gap" seen in this model.

Helper T-cells regulate inflammation. A lineage of these cells named Th17, distinct from both Th1 and Th2 lineages, is now recognized that is induced by interleukin (IL)-23 to produce IL-17 and other pro-inflammatory Th17 effector molecules [41]. The development, regulation and functional capacities of Th17 cells have recently been reviewed [42]. Dubin and colleagues have reported evidence for a central role of the IL-23/IL-17 pathway in the pathogenesis of human CF lung inflammation [15]. They found elevated protein levels of IL-17A and IL-17F during an acute pulmonary exacerbation of chronic *P. aeruginosa *lung infection in the sputum of adult CF patients, and in the BAL fluid of pediatric CF patients unable to produce sputum, levels that reduced dramatically following antibiotic treatment [14,15]. They also found that neutrophil recruitment in a murine model of *P. aeruginosa *lung infection is IL-23/IL-17 dependent [16]. Thus recent lines of evidence emphasize the correlation between levels of IL-23/IL-17 and the severity of lung inflammation in both CF mice and in the human disease.

In our CF mice with PA508 pneumonia, E2 increased protein levels in BAL fluid of TNFα and IL-6 (upstream stimulators of IL-17 production) and also increased mRNA levels in lung tissue of IL-23 and the isoforms IL-17A and IL-17F. We then confirmed increased BAL fluid protein content of IL-12(p40) (one of two heteromers comprising the IL-23 molecule) [16], IL-17A and downstream effector molecules: the pro-inflammatory IL-1α, MIP-1α, IL-6 and the IL-6 family member LIF, and chemoattractant chemokines that recruit macrophages (MCP-1), eosinophils (eotaxin and RANTES), and monocytes (M-CSF). E2 also increased the chemoattractant chemokines, MIP-2 (recruits neutrophils), MIP-1β (macrophages) and IL-15 (mast cells). There was no increase in the products of Th1 or Th2 cells that suppress the development of IL-17 producing T-cells, IFNγ and IL-4 respectively [41]. Together, these findings suggest that the mechanism of the observed pro-inflammatory effects of E2 in male CF mice involves stimulation of Th17 cells. Known or postulated intermediary interactions in pro-inflammatory stimulation by E2 are shown in additional file 1.

It remains controversial whether, in human CF lung, inflammation or infection precedes and initiates susceptibility to the other [43], although there is increasing recent evidence that the inflammatory response accounts for the majority of the morbidity and mortality of the human disease [3]. We contributed to a previous review of lung inflammation as a therapeutic target in CF [44]. Both inflammation and infection eventually become chronic and severe in CF lungs [1], each aggravating the severity of the other. A key feature of human CF lung disease - that this [13] and similar [12] mouse models of CF lung infection reproduce - is the marked increase in BAL fluid of inflammatory cells, predominately of neutrophils, that is not successful in clearing the *P. aeruginosa *infection [9-11]. Neutrophils particularly enhance the formation of the very antibiotic-resistant form of *P. aeruginosa*, biofilm [32]. IL-17 stimulates the expression of IL-8 and G-CSF in human airway epithelial cells [45]. In our present study, E2 increased both the proportion and absolute numbers of neutrophils in BAL fluid, consistent with the concept that E2 aggravates CF lung disease. Our data suggest the mechanism may involve E2 enhancement of stimulation of neutrophil production by the IL-17-responsive growth factor G-CSF, as well as E2-induced IL-17 mediated increases in levels of the chemoattractant murine IL-8 homolog (MIP-2) leading to increased recruitment of neutrophils to the site of inflammation.

Antimicrobial peptides are important parts of the innate immune system [46]. An exploratory study from Baltimore reported a positive correlation between the pulmonary function of CF patients as measured by the FEV_1 _and levels in their BAL fluid of mRNA encoding three specific antimicrobial peptides - LTF, PIP and statherin [47]. Statherin is a potent human antimicrobial peptide, but its mRNA was not detectable in mice. Therefore, we focussed only on LTF and PIP. LTF is active against *P. aeruginosa *[33]. It disrupts formation of biofilm, the most pathogenic form of *P. aeruginosa *in CF airways that is highly resistant to antibiotics. This is done by chelating iron, in turn preventing *P. aeruginosa *from accessing the iron it needs to make biofilm [33]. It has been argued it is likely that during *P. aeruginosa *-induced neutrophil necrosis, LTF (which is expressed in human airways) is digested by proteases of neutrophil and pseudomonal origin [32]. Thus, our finding of an E2-induced increase in the number and proportion of neutrophils in lung tissue and BAL fluid would be expected to reduce LTF protein levels. Combined with our demonstration of a greater than 99% reduction of LTF mRNA in lung tissue, the overall direct and indirect inhibition of LTF activity by E2 would be expected to substantially eliminate this component of the innate immune defence against *P. aeruginosa *in the airway. PIP is an antimicrobial peptide in the trachea, the expression of which has been also shown to correlate positively with lung function in CF patients. It is interesting to note that PIP mRNA levels in our CF mice were reduced by E2, but it is not known to what extent if any the relatively mild degree of reduction of PIP mRNA levels in the lungs of our mice would have any physiological effect on *P. aeruginosa*.

It is likely that E2 has more than one role in the pathogenesis of the gender gap in human CF disease. One possibility is that E2 further impairs the already deficient ability of CF airway epithelial cells to activate Cl^- ^secretion and thereby adequately hydrate the airway surface liquid. A widely held hypothesis to explain the predisposition of CF airways to chronic infection is that reduced volume of isotonic periciliary airway surface liquid impedes mucociliary clearance of pathogens and hypoxic mucus [48]. We [49] and others [30] have reported that normal menstrual changes in blood E2 levels are associated with changes in nasal potential differences, *in vivo *measures of fluid and electrolyte transport across the respiratory epithelium. Coakley and colleagues found that the high blood E2 levels around the time of ovulation are associated *in vivo *with an inhibition of UTP-mediated Cl^- ^secretion in females with CF [30]. Based upon studies of human bronchial epithelial cell culture, they found that this inhibition is due to changes in E2 concentration and not due to gender-based differences in estrogen receptor levels.

The finding that E2 did not induce an increase in the BAL fluid concentrations of some of the cytokines, chemokines and growth factors we assessed indicates that the demonstrated range of increases in concentrations of other inflammatory mediators is not simply an artefact of a reduced airway surface liquid volume due to an effect of E2 on transepithelial ion transport.

The association in E2-treated mice of increased signs of inflammation with higher bacterial burden in both the lung tissue and the BAL fluid illustrates the mutual reinforcement of infection and inflammation in this model. The anti-inflammatory IL-10, reported to be reduced in PA508 infection in CFTR knockout mice [13], was not altered in our present study. We speculate that it might have been reduced had we examined mice at a later and more florid stage in the pneumonia, but this awaits further investigation.

Regulation of IL-17 has recently been proposed as an attractive therapeutic approach for asthma [50], a common inflammatory respiratory condition involving eosinophilic and neutrophilic infiltrates that often co-exists in CF patients. A sex difference has long been recognized in asthma, with females in the minority as children but in the majority with more serious disease as adults (reviewed in [51]). Indeed, it is attractive to speculate that stimulation by E2 of Th17 pro-inflammatory signaling in the lung may contribute to the sex difference in asthma as well as in CF.

Circulating levels of progesterone, as well as of E2, are higher in sexually mature females than in males, with cyclical menstrual changes in progesterone and E2 levels, and in the ratio of progesterone to E2. The activity of cytokine-secreting cells in vivo correlates with sex hormone levels and fluctuates with menstruation [52]. Future study will be required to determine the effects of progesterone and of menstrual fluctuations in the ratio of circulating levels of progesterone to E2 on CF airway inflammation and infection.

Recently, Chotirmall et al. reported that E2 inhibits release by the human CF bronchial epithelial cell line CFBE41o- of the pro-inflammatory IL-8 by up-regulating secretory leucoprotease inhibitor [53]. While acknowledging that most published data in the field have thus far emphasized the damaging effects over time of uncontrolled chronic inflammation in CF lung, the authors propose that acute surges of inflammation may actually be protective in the context of an acute exacerbation. If confirmed, this would imply that E2 inhibition of IL-8 during acute exacerbations would be deleterious. This interesting suggestion remains to be tested in an intact animal or human - considering the possibility that effects of E2 upon a cell line *in vitro*, in the absence of cell-cell interactions, may not necessarily correspond to the effects on the whole, intact animal or human. Moreover, IL-8 is also produced by cell types other than airway epithelial cells, including neutrophils, of which there are large numbers in CF airway secretions (reviewed in [54]). The effects of E2 upon neutrophilic IL-8 production remain to be determined.

Although there is no exact murine homolog of IL-8, three closely related functional homologs (MIP-2, KC and LIX) have been identified in the mouse [40]. In our present study, E2 increased BAL fluid levels of the pro-inflammatory chemoattractant chemokine MIP-2, but levels of KC and LIX failed to reach statistical significance (p = 0.057). If, as our data suggest, increased inflammation in CF is harmful even in acute exacerbations, any anti-inflammatory activity of E2 would be expected to be beneficial and thus would not contribute to the pathogenesis of the observed CF gender gap. Alternatively, deleterious increases in pro-inflammatory mediators other than IL-8 and other effects of E2 (such as inhibition of aspects of innate immunity or mucociliary clearance) may more substantially aggravate CF lung disease over time than any benefit from reduction caused by E2 in IL-8 from airway epithelial cells during acute exacerbations, and thus E2 could make a net pro-inflammatory contribution to the cause of the CF gender gap. If E2 activity is harmful, E2 antagonists may hold therapeutic potential for CF whether that harmful activity is pro-inflammatory or anti-inflammatory. However, our present findings of increased inflammation in response to E2 suggest anti-inflammatory approaches may be beneficial.

We used the method of Guilbault *et al*. [13] to infect the lungs of our mice. To avoid the confounding effects of variability in circulating endogenous E2 levels associated with the female estrus cycle, we studied only adult male mice. Others using exactly the same strain of CF mice have reported a higher female mortality in response to acute exposure to intratracheal inoculation with PA508, and therefore only used male mice in their study [55]. Guilbault and colleagues have reported that both wild type [23] and CF [13] mice demonstrate a marked female disadvantage with respect to lung inflammation and infection. Using the same model system, instillation technique and PA508 clinical strain as in our present work, they reported CF mice displayed a marked female disadvantage in weight loss and mortality [13]. They too predominately used male mice in their studies due to high mortality in the females. Furthermore, the bacterial burden in lung tissue homogenates in females was between 30- and 300-fold higher than with the male mice. Yet, because of the high variability seen in the females, they found no statistical significance could be reached.

We examined our mice only two (rather than four) days after intratracheal injection of the PA508-impregnated agar beads in order to assess the degree of inflammation at an earlier (and therefore presumably milder) stage in the pathogenesis of the pneumonia, at a time when we reasoned it might be easier to detect E2-caused differences in inflammation. Our mice did not have any fatalities, unlike the mice with *P. aeruginosa *pneumonia described by the Montreal [23] or Cleveland [12] investigators, who were studied a later phase of the pneumonia. Our control group, at this earlier stage, displayed minimal evidence of lung tissue infiltration with inflammatory cells and negligible evidence of mucin, facilitating differentiation of the controls from the E2-treated group which showed infiltrates and mucin production.

## Conclusions

E2 treatment of adult male CF mice reproduced the known features of the "gender gap" that are present in an established model of CF PA508 lung infection. Pro-inflammatory Th17 mediators (molecules of the IL-23/IL-17 pathway) were increased and expression of antimicrobial peptides was inhibited, suggesting mechanisms of E2 action. Transferring these results to the human situation needs to be undertaken with caution, given the differences between the anatomy and physiology of the human and murine respiratory tracts and the differences between the immune systems of the two species. Moreover, male mice injected with E2 alone are not the equivalent of females. Nevertheless, the model reproduces important features of the human disease. Further investigation of the mechanism(s) of E2 effects on inflammation and infection with *P. aeruginosa *in the CF lung is justified to assess the potential therapeutic applicability in CF of the clinically available anti-estrogen compounds and of modulators of the Th17 pathway.

## List of abbreviations used

BAL: bronchial alveolar lavage; CF: cystic fibrosis; CFTR: CF transmembrane conductance regulator; CFUs: colony forming units; E2: 17β-estradiol; G-CSF: granulocyte colony-stimulating factor; GM-CSF: granulocyte-macrophage colony stimulating factor; H&E: hematoxylin and eosin; IFNγ: interferon gamma; IL: interleukin; IL-12 (p40): IL-12 sub-component p40; i.p.: intraperitoneally; IP-10: immune protein 10; KC: keratinocyte chemoattractant; LIF: leukemia inhibitory factor; LIX: lipopolysaccharide-inducible CXC chemokine; LTF: lactoferrin; MCP-1: monocyte chemotactic protein-1; M-CSF: macrophage colony-stimulating factor; MIG: monokine induced by interferon gamma; MIP: macrophage inflammatory protein; PAS: periodic acid Schiff; PIP: prolactin-inducible peptide; PMNs: polymorphonuclear cells; *P. aeruginosa: Pseudomonas aeruginosa; *RANTES: Regulated on Activation Normal T Cell Expressed and Secreted; Th: T helper cells; TLRs: toll-like receptors; TNFα: tumor necrosis factor alpha; VEGF: vascular endothelial growth factor; WBCs: white blood cells

## Competing interests

The authors declare that they have no competing interests.

## Authors' contributions

YW was primarily responsible for the conduct of the experiments and contributed to the study design, statistical analysis, preparation of the figures and interpretation of the results. EC assisted with the conduct of the experiments and statistical analysis. SG contributed to the conduct of the experiments, interpretation of the results and revision of the manuscript. NBS had primary responsibility for the study design, drafting and revising the manuscript, the statistical analysis and interpretation of the data, and participated in revisions of the figures. All authors read and approved the final manuscript.

## Supplementary Material

Additional file 1 **Conceptual network, showing known or postulated intermediary interactions in pro-inflammatory stimulation of CF mouse lung by E2**. PMNs (neutrophils), MΦ (macrophages), Eos (eosinophils). Click here for file
