# Supplementary material for: Estrogen aggravates inflammation in Pseudomonas aeruginosa pneumonia in cystic fibrosis mice
Source: Respir Res. 2010 Nov 30;11(1):166. doi: 10.1186/1465-9921-11-166 (PMC3006363; doi:10.1186/1465-9921-11-166)
Supplement: Additional file 1 — Conceptual network, showing known or postulated intermediary interactions in pro-inflammatory stimulation of CF mouse lung by E2. PMNs (neutrophils), MΦ (macrophages), Eos (eosinophils). [file 1465-9921-11-166-S1.PPT]

## Slide 1
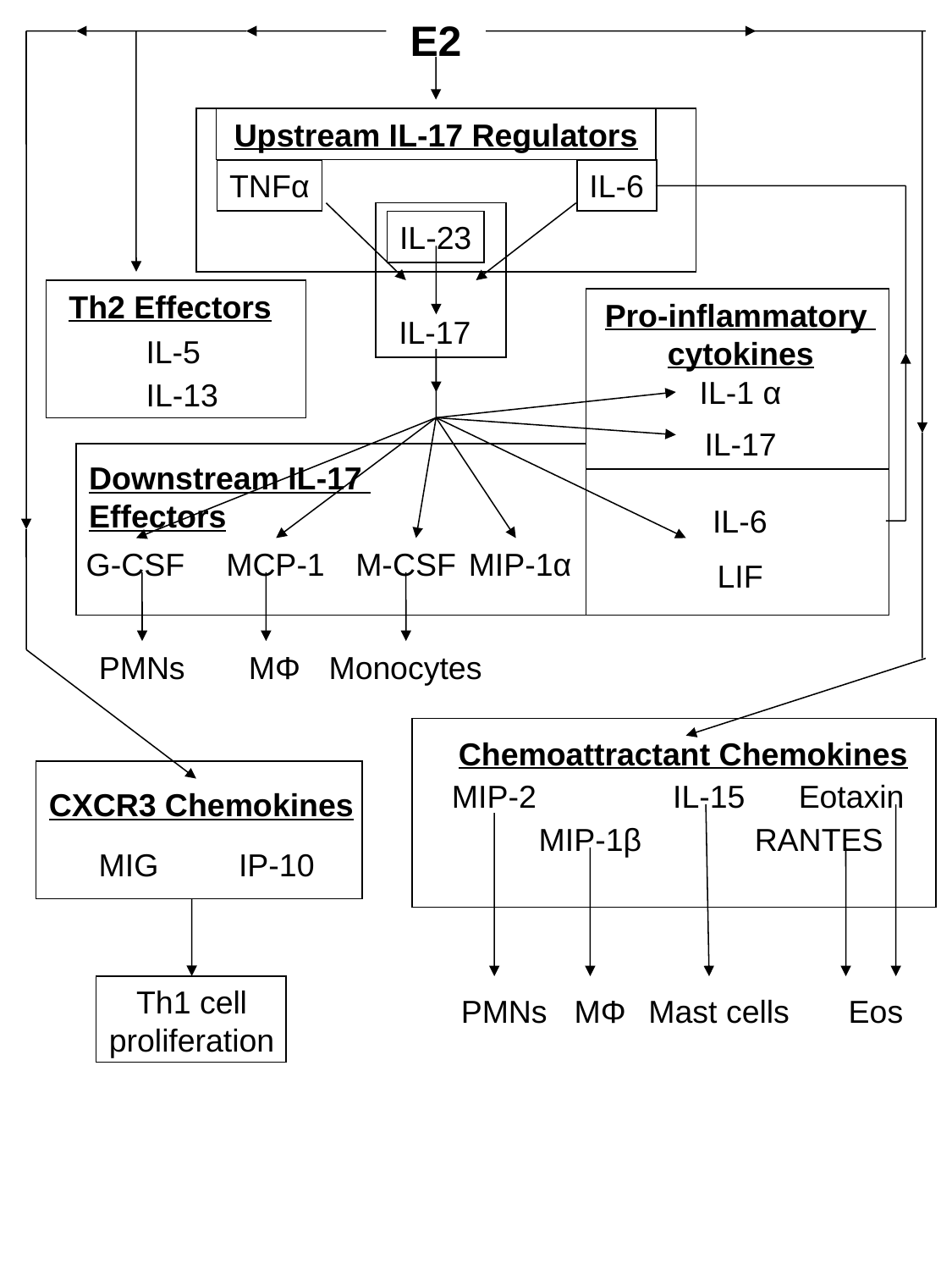

E2
Upstream IL-17 Regulators
TNFα
IL-6
IL-23
IL-17
Th2 Effectors
IL-5
IL-13
Pro-inflammatory
cytokines
IL-1 α
IL-17
IL-6
LIF
Downstream IL-17
Effectors
G-CSF
MCP-1
M-CSF
MIP-1α
PMNs
MΦ
Monocytes
Chemoattractant Chemokines
IL-15
MIP-2
Eotaxin
RANTES
MIP-1β
PMNs
MΦ
Mast cells
Eos
CXCR3 Chemokines
MIG
IP-10
Th1 cell
proliferation
